# Effect of breast cancer education based on the health belief model on knowledge, health belief, and breast self-examination among female students of Debre Markos University, Northwest Ethiopia, in 2021

**DOI:** 10.3389/fonc.2022.1034183

**Published:** 2022-11-09

**Authors:** Kumlachew Solomon Wondmu, Mekuanint Taddele Tessema, Genet Degu, Getachew Tilaye Mihiret, Melkam Tesfaye Sinshaw

**Affiliations:** ^1^ Department of Midwifery, College of Health Sciences, Debre Markos University, Debre Markos, Ethiopia; ^2^ Department of Public Health, College of Health Sciences, Injibara University, Injibara, Ethiopia

**Keywords:** health belief model, breast cancer, knowledge, health believes, breast self-examination, effect

## Abstract

**Background:**

Breast cancer is the primary cause of mortality in female patients around the world and the second most common cancer after lung cancer in both sexes. Socio-cultural factors contribute to the development, maintenance, and change of health behavior; knowledge, attitudes, and motivation are important individual determinants of health behavior change. Therefore, this study aims to evaluate the effect of health belief model-based breast cancer education on knowledge, health belief, and breast self-examination among female students of Debre Markos University, Ethiopia, in 2021.

**Methods:**

A quasi-experimental study was conducted on 210 samples selected by simple random sampling; samples were assigned to an intervention group and a control group based on their cluster. Data were collected by self-administration questionnaire before and after intervention and then entered into Epi data version 3.1 and analyzed by SPSS version 25. A dependent *t*-test and an independent *t*-test were used. Difference in difference was used to assess the effect of the intervention.

**Results:**

Statistically significant differences were observed between groups’ knowledge about breast cancer after educational intervention; monthly breast self-examination practice changes from 20.0% to 57.58% and 17.14% to 24.27% in intervention and control groups, respectively; and average scores of all health belief model constructs show a statistically significant change in the intervention group relative to controls after intervention. Overall knowledge increased by 6.24, perceived susceptibility increased by 4.67, perceived seriousness increased by 6.93, benefit of breast self-examination increased by 3.51, self-efficacy increased by 9.45, cues for action increased by 2.74, barriers of breast self-examination decreased by 3.61, and breast self-examination increased by 2.26 due to educational intervention.

**Conclusion:**

The health belief model-based education is an effective and efficient way to enhance students’ breast self-examination and promote knowledge and health beliefs about breast cancer. Based on the findings of this study, implementing a health belief model-based educational intervention about breast cancer at different phases of life is important to fight the disease.

## Introduction

Breast cancer (BC) is a disease that results from uncontrolled growth and changes in breast tissue, typically resulting in a lump or mass. It is the most common cancer and also the primary cause of mortality in female patients around the world (1). Globally, it is the leading cancer-related disease both in morbidity and in mortality among female patients, affecting 2.1 million annually, and over half million died in 2018. While BC rates are higher in more developed regions, it is increasing in every region internationally, including Ethiopia (2, 3).

In 2020, there were 2.3 million women diagnosed with BC and 685,000 deaths globally. At the end of 2020, there were 7.8 million women who were diagnosed with BC in the past 5 years. Low- and middle-income countries (LMICs) account for 57% and 65% of cancer cases and deaths, respectively. Diagnosed cases of BC showed a survival rate below 40% in developing countries and exceeding 80% in the developed world; these differences are due to their early health-seeking behavior. In Ethiopia, BC is the most prevalent cancer among women and constitutes a major public health concern, which accounts for 30% of all cancers (4–7).

The emergence of breast disease and the subsequent development of cancer tend to be more aggressive in young women compared to the older population and also have a worse prognosis. Although controversy exists about the definition of “very young age” or “very young patients” and different cutoffs have been proposed, it has been shown that younger age is associated with a less favorable prognosis and that the relationship between recurrence hazard and age was continuous with a 4% decrease in recurrence and a 2% decrease in cancer-specific death for every year of increase in age. In particular, in a recently published study, the risk of death increases by 5% for every 1-year reduction in age for patients aged <35 years, whereas there was no significant correlation between risk of death and age for patients aged 35–50 years (8–11).

The health belief model (HBM)-based education is an efficient way to raise people’s knowledge and subsequent behavioral change, which was developed by Hochbaum and Rosenstock in the 1950s. HBM was one of the earliest behavior change models to explain human health decision-making and subsequent behavior. It is a psychological model developed to explain why some people refused chest x-rays for detecting tuberculosis even though the service is free. What they discovered was that people’s beliefs about the severity of a disease and their susceptibility to it influenced their willingness to take preventive action. Over the next few years, this theory was modified to include six constructs to help predict whether people will take action to prevent, screen for, and control illness (12).

According to HBM, individuals should believe that even in the case of no symptom of disease, they might have it. When people see themselves at risk of a disease (perceived susceptibility), realize that a disease has potentially serious consequences (perceived severity), believe that preventive action has positive results (perceived benefits), believe that the barriers of that behavior is less than its acquired benefits (perceived barriers), and are confident in realizing that health behavior (self-efficiency), they are more likely to follow/maintain that behavior (13, 14).

In Ethiopia, BC becomes fatal due to late presentation; limited resources; low awareness of BC and its detection, symptoms, and prevention; and strong traditional beliefs that can delay biomedical care. As a result, many women miss early detection and treatment opportunities. According to a study done in the Tikur Anbessa Specialized Hospital, Ethiopia, among 16,622 new cancer cases registered, 3,460 (21.0%) were new BC cases, indicating about 216 cases annually. BC cases are among the top prevalent cases (31.5%), followed by cervical cancer, which accounts for 14% among women in the country (15).

Many social, cultural, and economic factors contribute to the development, maintenance, and change of health behavior patterns; knowledge, attitudes, and motivation are important individual determinants of health behavior change (16). Thus, the aim of this study is to improve knowledge, health belief, and BSE skills among female health science students.

## Methods and materials

### Study design, area, and period

A parallel cluster-randomized controlled trial (cRCT), with campuses being the unit of randomization, was used. The study was conducted at DMU, East Gojjam, Amhara regional state, Ethiopia from 15 April to 22 May 2021. Debre Markos University is found in the northwestern part of Ethiopia at Debre Markos town, which is located in the east, approximately 2 km from the central square covering an area of 100 ha. Debre Markos town is located 300 km northwest of Addis Ababa, which is the capital city of Ethiopia and 265 km southeast of Bahir Dar, the capital of the Amhara National Regional State. The university is founded in an area with immense research and investment potentials, suitable weather conditions, and tourist attraction sites. Its foundation stone was laid in January 2005 and initiated on 18 November 2007. After the completion of the first phase of the construction, the university began its service in February 2007, admitting the first batch of 760 regular students in the Education Faculty with 53 academics, 34 supporting permanent staff, and 21 contract workers (17).

### Population

All regular undergraduate female students of DMU main and Bure campus other than Medicine and Health Science College were the source population of the study, whereas the study population was composed of all regular undergraduate second-year female students of DMU main and Bure campus who are active in the study period in the selected departments.

### Sample size determination

The statistical software G*Power version 3.1 was used to estimate the required sample size applying the following parameters: a power of 0.80, a medium effect size of 0.5, a statistical significance of 0.05, and an allocation ratio of 1 between two groups, using independent-sample *t*-test, multiplied by 1.5 for multi-staging and 10% dropout. The final sample size was 210 [intervention group (*n* = 105) and control group (*n* = 105)].

### Sampling procedures

A multi-stage sampling method was used to select study participants first, two clusters were formed (i.e., main and Bure campus), and then two colleges were selected randomly (lottery method). In each of the selected colleges, three departments were selected randomly, and then proportional allocation was done for each selected department. Finally, the registration number of students was used as a sampling frame to select study participants. The reason for selecting second academic year students was that they did not have previous educational knowledge about BC or BSE, which gives a more representative result on the effect of the intervention. In addition, creating awareness at an earlier stage of life can have an enduring effect on young girls’ performance of BSE as a healthy habit throughout life.

### Study variables

#### Dependent variables

Knowledge about BC, and health beliefs about BC and BSE practice.

#### Independent variables

Socio-demographic characteristics (age, religion, college, department, family history of BC, and knowing a patient who had BC), participation in the training, and time.

#### Conceptual definition for constructs of HBM

##### Perceived susceptibility

It refers to personal beliefs about the likelihood of getting a disease or condition. For instance, a woman personally believes in the possibility of getting BC (18).

##### Perceived severity

It refers to feelings about the seriousness of contracting an illness or complication of the disease if not treated, as well as both medical and clinical consequences (for example, pain, disability, and death) and possible social consequences (such as effects on work, family life, and social relations in relation to getting BC) (18).

##### Perceived barriers

They refer to beliefs about the tangible and psychological costs of the advised action or negative aspects of a particular recommended health action (for example, belief that it may be expensive, it has negative side effects, and it is unpleasant, inconvenient, or time-consuming to perform BSE) (18).

##### Perceived benefits

They refer to personal beliefs regarding the efficacy of the advised/recommended action to reduce the risk of acquiring the illness or seriousness of the BC (18).

##### Cues for action/motivations

They refer to strategies/triggers to activate readiness to perform the advised action or to do BSE (18).

##### Self-efficacy

It refers to personal confidence in one’s ability to take the recommended action, in this case, BSE (18).

### Data collection tool and data quality control

#### Three tools were used for data collection

##### Tool I: Self-administrated questionnaire that includes the following parts:

**Part 1:** Socio-demographic data: age, religion, college, department, family history of BC, knowing a patient who had BC, and educational intervention.

**Part 2:** Knowledge assessment sheet: it was developed by the researcher in English language through reviewing relevant literature. This part was used before and after the implementation of the educational intervention in both groups. It includes 16 questions to assess second-year female students’ knowledge regarding BC, signs/symptoms, risk factors, and early screening methods (19).

##### Tool II: Health belief model scale

It was adapted from the refined version of the Champion Health Belief Model Scale (CHBMS) for BC (20). Modifications were done on the constructs of HBM by the researcher based on the result of Cronbach alpha (i.e., one question from perceived susceptibility, three from perceived benefits, two from perceived barriers, one from self-efficacy, and three from cues for action were removed). It was used to assess the students’ health beliefs about BC. The scale is composed of 32 items graded on a five-point Likert scale ranging from 1 (strongly disagree) to 5 (strongly agree) and scattered over six constructs as follows: perceived susceptibility (4 items) with the total score between 4 and 20; perceived severity (7 items) with the total score between 7 and 35; perceived benefits (3 items) with the total score between 3 and 15; perceived barriers (4 items) with the total score between 4 and 20; self-efficacy (10 items) with the total score between 10 and 50; and cues for action (4 items) with the total score between 4 and 20. The total HBM score was calculated by summing up the score of each construct, which created a total score that ranged from 32 to 160. The higher score indicates more positive beliefs toward BC and its preventive and screening behaviors except for barriers of BSE.

##### Tool III: BSE practice

It was used to assess students’ BSE practice. It was prepared by the investigator based on previous works (21). It is composed of nine items describing the practice, frequency, and examination technique of BSE.

The study was carried out through four phases: assessment, planning, implementation, and evaluation.

#### Assessment phase

Upon securing official approval to conduct the study, the researcher contacted students to collect pretest data on their demographic characteristics, knowledge regarding BC, health beliefs about BC, BCS, BSE practice, and HBM constructs. The data obtained during this phase were used as a baseline for further comparisons to assess the effect of the intervention.

#### Planning and implementation phase

Based on the gap identified in the assessment phase and given the related literature, the researcher has developed a PowerPoint presentation about BC based on HBM using simple English that suits the students’ level of understanding. It focused on major areas of deficiency regarding students’ knowledge about BC, which included anatomy and physiology of the breast, definition of BC, risk factors, signs and symptoms, screening methods for early detection of BC, and the BSE technique. The educational interventions involved two sessions that were conducted on a small group (25–40) of students. The duration of each session was from 01:30 to 02:30 h; the session was arranged based on their achievement, progress, and feedback. Different methods of teaching were used such as lecture, group discussion, brainstorming, and demonstration videos on BSE practice.

#### Evaluation phase

After education based on HBM, the effect of the educational intervention was evaluated by using the pretest questionnaire in both the intervention and the control group.

### Data quality control

All data collection tools of the study were reviewed by three experts in the field to ensure their clarity and applicability, and the tools were modified according to the experts’ comments on the simplicity of the questionnaire. A pilot study was done in Injibara University concerning 5% of the students (*n* = 12) to test the reliability of the study tools and to test the simplicity of the designed questionnaire and HBM scale.

Based on the pilot study results, Cronbach alpha was 0.721 for knowledge, 0.755 for perceived susceptibility by removing one item, 0.766 for perceived severity, 0.813 for the perceived benefit of BSE by deleting three items, 0.703 for perceived barriers of BSE by removing two items, 0.843 for self-efficacy, 0.723 by deleting three items, and 0.938 for BSE practice items. The training was given for data collectors and supervisors on the objective/purpose of the study, confidentiality of information, and respondents’ right to withdraw from the study if they are not comfortable. Daily checkups will be done by the investigators and supervisors for potential errors and data completeness during and after data collection.

### Data processing and analysis

Data were coded and entered into Epi data version 3.1 and then exported to SPSS Version 25 statistical software for analysis. Data cleaning was performed to check for frequencies, accuracy, consistencies, and missed values, and then descriptive analysis such as percentages, means, and measures of dispersion, tables, and texts were used to describe the data. Socio-demographic comparison between the intervention group and the control group was made by using chi-square and independent samples *t*-test. A dependent *t*-test and an independent *t*-test were used to test the significant differences within and between groups, and difference in difference was used to assess the effect of the intervention. The cutoff value for the statistically significant difference was considered at *p* < 0.05.

### Ethical consideration

Ethical approval was obtained before data collection from the DMU research ethical approval committee. Following an explanation of the purpose of the study, written consent was obtained from each participant. Also, they were informed that they are free to withdraw consent and discontinue participation without any form of prejudgments. Confidentiality of information and privacy of participants were assured for all the information provided.

## Results

### Socio-demographic characteristics of the respondents

A total of 210 study participants with a response rate of 100% were included at baseline. Due to six dropouts in the intervention group and two in the control group, the final response rate was 94.29% and 98% in the intervention group and the control group, respectively. The majority of participants were orthodox Christianity followers (80% and 67.6% for the experimental group and the control group, respectively). Both groups nearly have a similar mean age of 20.54 ± 1.0 and 20.36 ± 0.9 in the experimental and control group, respectively. Urban residency was prevalent in both the study group (57.1%) and the control group (54.3%). In addition, 58.1% of the study group and 57.1% of the control group were from the College of Business and Economics, of whom 34.3% and 26.7% were from the Department of Accounting for the study and control group, respectively; 6.7% of the study group and 11.4% of controls had a family history of BC; 13.3% of the study group and 18.1% of the control group know someone who had BC from their family. Most of the students in the intervention group (93.3%) and the control group (88.6%) had no family history of BC.

As illustrated in **Table 1**, there was no statistically significant difference between the study group and the control group based on their socio-demographic characteristics using independent *t*-test.

**Table 1:** Socio-demographic characteristics of intervention and control groups with comparison among female students of Debre Markos University, Amhara, Ethiopia before and 1 month after educational intervention about BC based on HBM (*n* = 210) 2021.

Variables	Intervention group		Control group		Statistics
	N	%	N	%
**Age in years**					
19	7	6.7	13	12.4	*t* = 1.38
20	57	54.3	56	53.3	p = 0.170
21	24	22.8	23	21.9	
22	13	12.4	11	10.5	
23 above	4	3.8	2	1.9	
**Religion**					
Orthodox Christian	84	80	71	67.6	*χ* ^2^ = 4.920
Muslim	5	4.7	12	11.4	p = 0.085
Protestant	16	15.3	22	21	
**Residence**					
Urban	60	57.1	57	54.3	*χ* ^2^ = 0.174
Rural	42	42.9	48	45.7	p = 0.677
**College of participants**					
Agriculture	44	41.9	45	42.9	*χ* ^2^ = 0.020
Business and economics	61	58.1	60	57.1	p = 0.889
**Department participants**					
Horticulture	12	11.4	13	12.4	*χ* ^2^ = 3.13
Management	16	15.2	17	16.2	p = 0.679
Agro-economics	17	16.2	14	13.3	
Accounting	36	34.3	28	26.7	
Economics	9	8.6	15	14.3	
NARM	15	14.3	18	17.1	
**Family history of BC**					
Yes					χ^2^ = 1.45
No	98	93.3	93	88.6	p = 0.067
**Who has breast cancer in the family**					
Mother	2	28.6	7	58.3	
Sister	3	42.8	2	16.7	
Grandmother	1	14.3			
Aunt	1	14.3	3	25	
**Knowing someone who has breast cancer**					
Yes	14	13.3	19	18.1	*χ* ^2^ = 0.899 p = 0.343
No	91	86.7	86	81.9	

### Knowledge of participants about BC

Study results show that there is no statistically significant difference between the intervention group and the control group’s mean total knowledge, risk factors of BC, signs/symptoms of BC, and screening methods of BC score before educational intervention. On the other hand, after educational intervention based on HBM, a statistically significant difference was detected between the study group and the control groups’ mean total knowledge score. Mean total knowledge score changes from 6.54 ± 2.86 to 13.0 ± 2.71 and from 6.33 ± 3.16 to 6.54 ± 3.16 in the intervention group and the control group, respectively. Additionally, there was a general improvement in knowledge mean scores within the intervention group after 1-month educational intervention as compared to before (*p* = 0.00*). However, no statistically significant differences were observed within the control group. The findings can be seen in **Table 2**.

**Table 2:** Mean differences between the intervention and control groups’ knowledge score regarding breast cancer among female students of Debre Markos University, Amhara, Ethiopia before and 1 month after educational intervention about BC based on HBM (n = 210) 2021.

	Before intervention			One month after intervention		
Knowledge about breast cancer						
maximum score	intervention group	control group		intervention group	control group	
	(n= 105)	(n=105)		(n= 99)	(n=103)	
	mean ± SD	mean ± SD	p- value	mean ± SD	mean ± SD	p-value
knowledge about BC risk factors(7)	2.87 ± 1.55	2.71 ± 1.57	0.48	5.78 ± 1.45	2.92 ± 1.67	0.00*
Significance within group	Intervention group: t =13.07 (p= 0.00*)					
	Control group: t = 1.19 (p = 0.239)					
knowledge about sign/symptoms of BC (6)	2.19 ±1.41	2.28 ± 1.57	0.678	4.85 ± 1.15	2.40 ± 1.68	0.00*
Significance within group	Intervention group: t = 13.36 (p= 0.00*)					
	Control group: t = 0.68 (p = 0.497)		
Knowledge about BC screening methods(3)						
	1.49 ± 0.98	1.34 ± 0.97	0.29	2.37 ± 0.85	1.22 ± 0.98	0.00*
Significance within group	Intervention group: t = 6.53 (p= 0.00*)					
	Control group: t = - 0.91 (p = 0.366)		
Total knowledge score(16)	6.54 ± 2.86	6.33 ± 3.16	0.615	13.0 ± 2.71	6.54 ± 3.16	0.00*
Significance within group	Intervention group: t = 14.68 (p= 0.00*)					
	Control group: t = 0.71(p = 0.480)		

*p - value < 0.001.

### Health belief participants about BC

The findings of the current study show that there are no statistically significant differences between the study group and the control group on perceived susceptibility (*p* = 0.614), perceived severity (*p* = 0.059), perceived benefits (*p* = 0.608), perceived barriers (*p* = 0.496), perceived self-efficacy (*p* = 0.165), and cues/motivation (*p* = 0.131) at baseline, but after the educational intervention, highly statistically significant differences (*p* = 0.00*) were observed between the intervention group and the control group in each construct of HBM. The overall mean of HBM changes from 85.29 ± 11.85 to 104.27 ± 11.57 and from 82.36 ± 15.45 to 77.65 ± 14.33 in the intervention group and the control group, respectively, after the educational intervention. Additionally, there was a general improvement in all HBM constructs’ mean scores within the intervention group 1 month after educational intervention as compared to before it (*p* = 0.00*). However, no statistically significant differences were observed within the control groups except overall HBM, which shows a significant change. **Table 3** shows the overall result.

**Table 3:** Mean differences between the study and control groups regarding HBM constructs among female students of Debre Markos University, Amhara, Ethiopia before and 1 month after educational intervention about BC based on HBM (n = 210) 2021.

Constructs of HBM	Before intervention			One month after intervention		
maximum score	intervention group (n= 105) mean ± SD	control group (n=105) mean ± SD	p-value	intervention group (n=99) mean ± SD	control group (n=103) mean ± SD	p-value
Perceived susceptibility (20)	7.56 ± 3.03	8.04 ±3.20	0.269	11.76 ± 2.89	7.56 ± 2.88	0.00*
Significance within group	Intervention group: t = 10.31 (p= 0.00*)					
	Control group: t = - 1.11 (p = 0.270)					
Perceived seriousness(35)	19.10 ± 5.36	17.57 ± 6.24	0.059	24.79 ± 4.20	15.77 ± 5.54	0.00*
Significance within group	Study group: t = 8.46(p= 0.00*)					
	Control group: t = - 1.40(p = 0.166)		
Perceived benefits (15)	7.86 ± 2.29	7.69 ± 2.55	0.608	10.98 ± 2.09	7.30 ± 2.52	0.00*
Significance within group	Intervention group: t = 10.00 (p= 0.00*)					
	Control group: t = - 0.97(p = 0.337)		
Perceived barriers (20)	12.54 ± 2.52	12.84 ± 3.65	0.496	8.67 ± 2.79	12.57 ±3.73	0.00*
Significance within group	Intervention group: t = -11.77 (p= 0.00*)					
	Control group: t = -0.65(p = 0.520)		
Self-efficacy (50)	26.21 ± 6.74	24.99 ± 5.90	0.165	33.84 ± 5.92	22.37 ± 6.52	0.00*
Significance within group	Intervention group: t = 8.11 (p = 0.00*)					
	Control group: t – 1.94(p = 0.055)		
Cues to action/motivation (20)	12.02 ± 3.86	11.24 ± 3.61	0.131	14.24 ± 3.02	10.72 ± 3.74	0.00*
Significance within group	Intervention group: t = 5.06 (p = 0.00*)					
	Control group: t = - 0.85(p = 0.397)		
HBM total	85.29 ± 11.85	82.36 ± 15.45	0.125	104.27 ± 11.57	77.65± 14.33	0.00*
Significance within group	Intervention group: t = 10.66(p = 0.00*)					
	Control group: t = - 2.10(p = 0.042)		

*p - value < 0.001.

### BSE practice of participants

The findings of this study revealed that there is no statistically significant difference between the intervention group and the control group’s BSE practice before educational intervention. Nevertheless, after educational intervention, a statistically significant difference was observed between the two groups’ BSE practice, which is higher in the intervention group than the control. Moreover, there was a statistically significant difference in BSE practice in the intervention group after 1-month educational intervention; conversely, the difference was insignificant in the control group. BSE practice per month changes from 23.89% to 57.41% (*p* = 0.00*) and from 17.86% to 24.55% (*p* = 0.264) for the intervention group and the control group, respectively, as **Table 4** shows.

**Table 4:** Mean differences between the intervention and control groups regarding BSE practice score among female students of Debre Markos University, Amhara, Ethiopia before and 1 month after educational intervention about BC based on HBM (n = 210) 2021.

BSE practice	Before intervention			One month after intervention		
		intervention group	control group	p-value	intervention group	control group	p-value
		(n= 105)	(n=105)			(n=99)		(n=103)	
		%± SD	% ± SD			%± SD		%± SD	
BSE practice in the last one month	23.81% ±0.43	17.14% ± 0.38	0.233	58.59 ± 0.5	24.27% ± 0.43	0.00*
Significance within group	Intervention group: t = 5.90 (p=0.00*)					
	Control group: t = 1.34 (p = 0.184)					
BSE practice between day 7-10 after menses before intervention	18.10% ± 0.39	20.0% ± 0.40	0.727	48.48% ± 0.50	25.24% ±0.44	0.001
Significance within group	Intervention group: t = 4.14 (p= 0.00*)					
	Control group: t = 1.06 (p = 0.291)					
BSE practice in front of mirror after intervention	24.76% ± 0.43	16.19% ± 0.37	0.125	48.48% ± 0.5	23.30% ± 0.42	0.00*
Significance within group	Intervention group: t = 3.73 (p= 0.00*)					
	Control group: t = 1.34(p = 0.184)					
Observation of unusual shape and size change during BSE	33.33% ±0.47	37.14% ± 0.49	0.566	69.70% ± 0.46	45.63% ± 0.50	0.00*
Significance within group	Intervention group: t = 5.60 (p= 0.00*)					
	Control group: t = 1.22 (p = 0.227)					
BSE once per month	20.0% ± 0.40	17.14% ± 0.38	0.597	57.58 ± 0.5	24.27% ± 0.43	0.00*
Significance within group	Intervention group: t=5.45 (p = 0.00*)					
	Control group: t = 1.34(p = 0.184)					
Use of vertical and circular technique during BSE	27.62% ± 0.45	19.05% ± 0.39	0.143	61.62% ± 0.49	27.18% ± 0.45	0.00*
Significance within group	Intervention group: t = 5.61(p = 0.00*)					
	Control group: t = 1.41(p = 0.161)					
Pressing of nipple to check unusual discharge during BSE	22.86% ± 0.42	22.86% ± 0.42	1	65.66% ± 0.48	26.21% ± 0.44	0.00*
Significance within group	Intervention group: t = 6.59 (P = 0.00*)					
	Control group: t = 0.65 (p = 0.519)					
Checking of armpit during BSE	31.43% ± 0.47	20.95% ± 0.41	0.085	54.55% ± 0.5	28.16% ± 0.45	0.00*
Significance within group	Intervention group: t = 3.50 (P = 0.001)					
	Control group: t = 1.30(p = 0.196)					
Rising of hands above the head during BSE	31.43% ± 0.47	20.95 ± 0.40	0.085	52.53% ± 0.51	25.24% ± 0.43	0.00*
Significance within group	Intervention group: t = 3.28 (P = 0.001)					
	Control group: t = 0.821(p = 0.414)					

*p - value < 0.001.

### The general effect of educational intervention on knowledge, health belief, and BSE practice

The intervention based on the HBM has a significant effect on knowledge about BC, on each construct of HBM and BSE practice. The findings of this study show that overall knowledge of the intervention group increased by 6.24 as a result of educational intervention.

The findings of the current study revealed that perceived susceptibility increased by 4.67, perceived seriousness increased by 6.93, the benefit of BSE increased by 3.51, self-efficacy increased by 9.45, cues for action increased by 2.74, and perceived barriers of BSE decreased by 3.61 due to educational intervention. Furthermore, practice of BSE increased by 2.26 as a result of educational intervention, as shown in **Table 5**.

**Table 5:** Difference in difference about total knowledge, health belief, and BSE practice score among female students of Debre Markos University, Amhara, Ethiopia before and 1 month after educational intervention about BC based on HBM (*n* = 210) 2021.

Variable	Coefficients	Std. Err	*t*	*p*-value	95% CI
**Knowledge**					
Time Training Time*training Cons	0. 21 0.21 6.24 6.33	0.41 0.41 0.59 0.29	0.51 0.51 10.63 21.76	0.611 0.611 0.000 0.000	−0.602–1.02 −0.599–1.01 5.09–7.40 5.76–6.90
**Susceptibility**					
Time Training Time*training Cons	−0.47 −0.48 4.67 8.04	0.42 0.41 0.59 0.29	−1.14 −1.15 7.89 27.42	0.255 0.251 0.000 0.000	−1.29–0.34 −1.29–0.33 3.51–5.83 7.46–8.61
**Seriousness**					
Time Training Time*training Cons	−1.24 1.52 6.93 17.57	0.73 0.73 1.04 0.52	−1.69 2.09 6.64 34.01	0.092 0.038 0.000 0.000	−2.68–0.20 0.88–2.96 4.88–8.99 16.56–18.59
**Benefit of BSE**					
Time Training Time*training Cons	−0.38 0.17 3.51 7.69	0.33 0.33 0.47 0.23	−1.17 0.52 7.51 33.22	0.243 0.601 0.000 0.000	−1.03–0.26 −0.47–0.81 2.59–4.43 7.23–8.14
**Barriers of BSE**					
Time Training Time*training Cons	−0.27 −0.30 −3.61 12.84	0.45 0.44 0.63 0.31	−0.59 −0.66 −5.69 40.87	0.553 0.507 0.000 0.000	−1.14–0.61 −1.17–0.58 −4.86–(−2.36) 12.22–13.46
**Self-efficacy**					
Time Training Time*training Cons	−1.83 1.22 9.45 24.99	0.86 0.85 1.22 0.60	−2.13 1.43 7.76 41.41	0.034 0.154 0.000 0.000	−3.51–(−0.14) −0.46–2.90 7.06–11.85 23.80–26.18
**Cues for action**					
Time Training Time*training Cons	−0.52 0.78 2.74 11.24	0.50 0.49 0.71 0.35	−1.05 1.58 3.89 32.18	0.296 0.115 0.000 0.000	−1.50–0.46 −0.19–1.75 1.36–4.13 10.55–11.92
**BSE practice**					
Time Training Time*training Cons	0.58 0.42 2.26 1.91	0.35 0.35 0.50 0.25	1.65 1.20 4.51 7.73	0.099 0.232 0.000 0.000	−0.11–1.27 −0.27–1.11 1.27–3.24 1.43–2.40

*Shows that the interaction/multiplication effect of time and training on the outcome variable.

## Discussion

The results of the current study indicate the significance of health education based on HBM in improving students’ knowledge about BC, regarding risk factors, signs/symptoms, screening methods, and BSE practice.

The results of the current study revealed a statistically significant positive modification in the mean score of total BC knowledge, risk factors, signs/symptoms, and screening methods of BC after educational intervention in the study group relative to the control group and within the study after the educational intervention, which is similar to a single-group pretest–posttest study done among healthy women in Turkey (22), a single-subject pre–post-study done in India (23), and a single-group, pretest–posttest study done in China (24). Also, it is similar to a study done in Iran (25), a study done in Jordan (3), a pretest–posttest control group study done based on HBM in Egypt (26), and two single-group pretest–posttest studies conducted in Egypt (27, 28).

The findings of this study showed no statistically significant difference in perceived susceptibility before educational intervention between the study group and the control group, but a statistical significance was found after intervention between the two groups (*p* = 0.000), and a statistically significant change was also seen within the intervention group after the educational intervention (*p* = 0.000), but not in the control group. This is similar with a pre-post control group study conducted based on the HBM in Iran (22, 26), and also with other study done in Iran among women 20–60 years old from based on HBM (27).

However, it is different from a study done in Turkey, which is insignificant on all constructs of HBM (29); the possible reason for this difference might be the time difference given in the training since the time used in Turkey was 90 min and it also has a small sample size (48). It is also different from a single-group pre/post study done in China (24); the possible reason for this variation might be the different family history of BC since the family history of BC in this study was seven and only one in China, which affects BC perceived susceptibility.

The findings of the current study show that there was no statistically significant difference in perceived severity between the two groups at baseline. A statistically significant difference was not found within the control group after the intervention even if a statistically significant difference was seen in the experimental group (p = 0.000). This is similar to a study conducted in Iran (30) to examine the effects of an educational intervention based on HBM on BSE behavior among women referred to health centers (25).

However, it is different from a study done in Turkey, which is insignificant on all constructs of HBM (29); the possible reason for this difference may be the time difference given for the training since the Turkey study gave 90 min for the educational intervention, had a small sample size, and did not assess the level of BC history.

The current study revealed no statistically significant difference at baseline between the intervention group and the control group in perceived benefit (*p* = 0.608), but after educational intervention, there was a statistically significant difference between the two groups (*p* = 0.000); additionally, there was a statistically significant improvement within the intervention group (*p* = 0.000) but not in the control group (*p* = 0.337). This finding was similar to a study done in Iran among health volunteers (25, 30, 31) and a study done in Egypt among nursing students to assess knowledge, health beliefs, and BSE practice based on HBM (26). However, it is different from a study done in Turkey, which is insignificant on all constructs of HBM (29). The difference might be the small sample size used in the Turkey study and the family history of BC, since the findings in the Turkey study did not assess the level of BC history.

The findings of this study show no statistically significant difference regarding perceived barriers of BSE in the pre-intervention period between the two groups; conversely, a statistically significant difference was established after educational intervention (*p* = 0.000). Also, a statistically significant difference was seen within the intervention group after the intervention (*p* = 0.000). On the other hand, there was no significant change within the control group after the educational intervention, which is similar to a study done in Egypt (26) and Iran (25, 30, 31). However, it is different from a study done in Turkey based on paired *t*-test, which is insignificant on all constructs of HBM (29), and a study done in China (24). The reason for this difference may be the small sample size used in China (60) and Turkey (48).

The findings of this study show no statistically significant difference regarding self-efficacy of BSE at baseline between the intervention group and the control group (*p* = 0.165); on the other hand, a statistically significant difference was found between groups after educational intervention (*p* = 0.000); this difference was also seen within the intervention group but not in controls post-intervention, which is similar to a study done in Iran (25, 30, 31), and to a study done in Egypt among nursing students to assess knowledge, health beliefs, and BSE practice based on HBM (26). However, it is different from a study done in Turkey, which is insignificant on all constructs of HBM (29). The possible reason for this variation may be the sample size difference and the short training time in the Turkey study.

The findings of this study show no statistically significant difference in cues to action before educational intervention between the study group and the control group (*p* = 0.131), but a statistically significant difference was found after intervention between the two groups (*p* = 0.000) and also a statistically significant change was seen within the intervention group after the intervention (*p* = 0.000), but not in the control group. This is similar to a pre–post control group study conducted in Iran, which was statistically significant immediately and 2 months after intervention (25, 30, 31) and a study done in Egypt among nursing students to assess knowledge, health beliefs, and BSE practice based on HBM (26). However, it is different from a study done in Turkey, which is insignificant on all constructs of HBM (29). The reason for this difference might be the sample size difference.

Breast self-examination is the easiest and most efficient way for BC prevention and early detection. The findings of this study show that the mean score of BSE practice has a statistically significant difference in the study group compared to the control group after educational intervention and within the study group before and after the educational intervention. This finding was similar to a study in Iran (25, 32), to a true experimental pretest–posttest study conducted in Jordan (33), to a study done in Nigeria among adolescent girls (34), and to a study conducted in Hawassa Health Sciences College Ethiopia first-year female midwifery students (35).

However, it is different from a study conducted in Nigeria assessing the impact of education on knowledge, attitude, and practice of breast self-examination among adolescent girls (36). The results in Nigeria show a significant decrease in BSE practice in the intervention group, but is constant in the control group. The possible reason for this difference might be a difference in the study population since most of the participants in the Nigerian study were between 14 and 16 years old. Thus, after intervention, most of the participants know the starting age of BSE; as a result, they will reduce BSE practice until they reach the recommended age.

### Limitations of the study

The study outcomes are evaluated based on self-reported information; this may lead to underestimation and/or overestimation due to social desirability. Another limitation is the lack of using an observational checklist of BSE tool to measure BSE practice.

## Conclusions

Based on the findings of the current study, the study concluded that HBM-based education is an effective and efficient way to enhance students’ breast self-examination practice and promote their knowledge level and health beliefs about BC. It is vitally important that students should be educated to improve their awareness of breast health. In general, the intervention helps in improving perceived susceptibility, the severity of BC, perceived benefits, and perceived self-efficacy with decreased barriers of BSE.

## Recommendations

➢ Arrange training sessions on BSE in collaboration with the education bureau and other NGOs through focusing on improving knowledge on BSE and skills on how to perform BSE.
➢ Use local social media as a means of disseminating information on BSE practice to improve the knowledge of BC, skills on how to perform BSE, confidence, and motivation, and minimize the barriers of BSE practice through inviting well-educated professionals.

### For researchers

➢ Repetition of this study by including an observational checklist of BSE is recommended for researchers to address BSE practice skills objectively.

### For health professionals

➢ Health extension workers and other health professionals ought to be encouraged to teach about BSE and its techniques in a different setting.

### For the ministry of health

➢ Organize HBM-based educational intervention about BC at a different phase of life and sites to reach all women.
➢ Develop instructional leaflets about BC based on HBM on different areas to improve knowledge, health beliefs, and BSE practices.
➢ Work in collaboration with the Ministry of Education to address the community at large by using different media platforms to prevent late presentation of BC cases to health facilities and reduce the maternal morbidity and mortality related to late health-seeking behavior.

## Data availability statement

The original contributions presented in the study are included in the article. Further inquiries can be directed to the corresponding author.

## Ethics statement

The studies involving human participants were reviewed and approved by Debre Markos University research ethical approval committee. The patients/participants provided their written informed consent to participate in this study.

## Author contributions

KW selected the title, wrote the proposal (conceptualized and designed the study), facilitated the data collection, analyzed and interpreted the data, and drafted the manuscript. MS approved the title, the proposal, and thesis with some revisions. GD approved the proposal and thesis with some revisions. GM participated in developing tools and data analysis, and revised drafts of the paper. MT participated in developing tools, data analysis, and report write up, and read and revised drafts of the paper. All authors contributed to the article and approved the submitted version.

## Acknowledgments

First, our gratitude goes to Debre Markos University for giving us the chance to study this program. Second, we would like to extend our thanks to study participants for giving the necessary information for the study and also data collectors and supervisors for their work. Third, we would like to thank our friends and colleagues for their support and friendly advice. Last, but not least, we extend our thanks to all institutions and individuals who helped us throughout this study.

## Conflict of interest

The authors declare that the research was conducted in the absence of any commercial or financial relationships that could be construed as a potential conflict of interest.

## Publisher’s note

All claims expressed in this article are solely those of the authors and do not necessarily represent those of their affiliated organizations, or those of the publisher, the editors and the reviewers. Any product that may be evaluated in this article, or claim that may be made by its manufacturer, is not guaranteed or endorsed by the publisher.

## Abbreviations

ACS, American Cancer Society; BC, Breast Cancer; BCS, Breast Cancer Screening; BSE, Breast Self-Examination; CBE, Clinical Breast Examination; DMU, Debre Markos University; HBM, Health Belief Model; KAP, Knowledge, Attitude and Practice; LMICs, Low- and Middle-Income Countries; UOG, University of Gondar; WHO, World Health Organization.
